# Genome Sequence of Arthrobacter sp. UKPF54-2, a Plant Growth-Promoting Rhizobacterial Strain Isolated from Paddy Soil

**DOI:** 10.1128/MRA.01005-19

**Published:** 2019-11-07

**Authors:** Weishou Shen, Xinchun Yu, Nan Gao, Sayuri Ota, Yutaka Shiratori, Tomoyasu Nishizawa, Kazuo Isobe, Xinhua He, Keishi Senoo

**Affiliations:** a Jiangsu Key Laboratory of Atmospheric Environment Monitoring and Pollution Control, Collaborative Innovation Center of Atmospheric Environment and Equipment Technology, and School of Environmental Science and Engineering, Nanjing University of Information Science and Technology, Nanjing, China; b National Engineering Research Center for Biotechnology and School of Biological and Pharmaceutical Engineering, Nanjing Tech University, Nanjing, China; c Niigata Agricultural Research Institute, Niigata, Japan; d Department of Bioresource Science, College of Agriculture, Ibaraki University, Ibaraki, Japan; e Department of Applied Biological Chemistry, Graduate School of Agricultural and Life Sciences, The University of Tokyo, Tokyo, Japan; f Centre of Excellence for Soil Biology, College of Resources and Environment, Southwest University, Chongqing, China; g Collaborative Research Institute for Innovative Microbiology, The University of Tokyo, Tokyo, Japan; University of Maryland School of Medicine

## Abstract

Arthrobacter sp. strain UKPF54-2, a plant growth-promoting rhizobacterium having the potential ability to control fungal and bacterial pathogens, was isolated from paddy soil in Kumamoto, Japan. We report here the whole-genome sequence of this strain.

## ANNOUNCEMENT

Arthrobacter sp. strains are a group of rod-shaped or coccoid Gram-positive bacteria in soil that can grow under aerobic and anaerobic conditions (1). Some *Arthrobacter* sp. strains are able to promote plant growth (1–4), enhance heavy metal phytoextraction (5–7), degrade organic and polyaromatic pollutants (8, 9), and restrain plant-pathogenic bacteria and fungi (1, 3, 10). Results from this present study have shown that *Arthrobacter* sp. strain UKPF54-2, originally isolated from the rhizosphere of paddy soil in Kumamoto, Japan (11), can promote the growth of a number of vegetable crops, such as komatusna (Brassica rapa L., Brassicaceae), crown daisy (Glebionis coronaria L., Asteraceae), parsley (Petroselinum crispum [Mill.] Fuss, Apiaceae), baby carrot (Daucus carota L., Apiaceae), and radish (Raphanus sativus L., Brassicaceae).

A single colony of *Arthrobacter* sp. strain UKPF54-2 was cultured in 5-ml nutrient broth culture medium containing 0.3 mM NaNO_3_ and 4 mM sodium succinate (pH 7.0) for 2 to 3 days and incubated at 26°C with shaking at 220 rpm. The genomic DNA was extracted with a DNeasy blood and tissue kit (Qiagen, Germany). The template prep kit 1.0 and BluePippin size selection system were used to construct a SMRTbell library with a 20-kb insert size. Whole-genome sequencing was performed on the PacBio RS II DNA sequencing system using C4 chemistry. A total of 1,623,497,927 bases were obtained. The mean subread length was 9,026 bp. The *N*_50_ value of the raw sequences is 11,645 bp. Up to 179,866 reads were obtained by filtration. The Falcon software (v 0.2.1) (12) was used to assemble the PacBio long reads with default parameters, except for daligner-selected overlap detection and error correction of raw reads. One contig was acquired accordingly. The ends of this contig are overlapped to generate a single circular contig for the chromosome. *Arthrobacter* sp. strain UKPF54-2, with a depth of about 260×, has a circular chromosome size of 3,517,818 bp, with a G+C content of 68.5%.

A total of 3,110 protein-coding sequences, 50 tRNAs, 15 rRNAs, 3 noncoding RNAs (ncRNAs), and 60 pseudogenes were discovered using the NCBI Prokaryotic Genome Annotation Pipeline (PGAP; revision 4.8) with the best-placed reference protein set (GeneMarks-2+) with default parameters (13, 14). Results from BlastKOALA (15) against the species_prokaryotes database with default parameters showed that the annotated functional genes consisted of 155 genes of the transporters, 31 genes of the secretion system, 10 genes of the two-component system, and 30 genes of the bacterial motility proteins. These predicted genes contained candidate genes relevant to plant growth promotion (Table 1). They also contained genes encoding acetolactate synthase (*ilvCDN*), which is involved in the synthesis of the volatile plant growth promotion signaling molecule acetoin. Furthermore, they contained 5 genes related to antimicrobial resistance.

**TABLE 1:** Predicted genes relevant to plant growth promotion in the *Arthrobacter* sp. strain UKPF54-2 genome

Gene function	Gene name	Product	GenBank accession no.
Nitrogen fixation	*nifU*	SUF system NifU family Fe-S cluster assembly protein	WP_017199296
Acetolactate synthase production	*ilvC*	Ketol acid reductoisomerase	WP_013493182
	*ilvD*	Dihydroxy acid dehydratase	WP_011692381
	*ilvN*	Acetolactate synthase small subunit	WP_015937457

This report will allow for genome-wide comparative analysis among *Arthrobacter* species or between this strain and other plant growth-promoting rhizobacterial strains that will provide fundamental support for developing biofertilizer.

### Data availability.

This whole-genome sequence of *Arthrobacter* sp. strain UKPF54-2 is available at GenBank under the accession number CP040174. The raw reads have been deposited in the Sequence Read Archive (SRA) under the accession number SRR8929632.
